# Locus Coeruleus Optogenetic Modulation: Lessons Learned from Temporal Patterns

**DOI:** 10.3390/brainsci11121624

**Published:** 2021-12-09

**Authors:** Carolyn W. Harley, Qi Yuan

**Affiliations:** 1 Psychology Department, Faculty of Science, Memorial University of Newfoundland, St. John’s, NL A1B 3X9, Canada; 2 Biomedical Sciences Department, Faculty of Medicine, Memorial University of Newfoundland, St. John’s, NL A1B 3V6, Canada; Qi.Yuan@med.mun.ca

**Keywords:** locus coeruleus, noradrenaline, olfactory learning, stress, arousal

## Abstract

After reviewing seminal studies using optogenetics to interrogate the functional role of the locus coeruleus in behavior, we conclude that differences in firing rates and firing patterns of locus coeruleus neurons contribute to locus coeruleus nucleus heterogeneity by recruiting different output circuitry, and differentially modifying behavior. The outcomes initiated by different optogenetic input activation patterns and frequencies can have opposite consequences for behavior, activate different neurons in the same target structure, be supported by distinct adrenoceptors and vary with behavioral state.

## 1. Introduction

The initial descriptions of locus coeruleus (LC) neuron firing in awake rats highlighted two patterns. The first was tonic discharges that reflected different phases of the sleep–wake cycle, with rates between 1–4 Hz in waking, slowing to 0.5 Hz in slow wave sleep and being nearly absent in rapid eye movement sleep. The second was brief phasic discharges of 5–10 Hz that accompanied spontaneous or sensory-evoked interruptions of on-going behavior [1,2]. Phasic bursts were typically followed by transient silence. Later studies in behaving rat supported a link between phasic LC firing and novelty [3,4] that rapidly habituated as well as revealing selective phasic responses to contingency changes.

Behavioral studies in monkeys evaluating tonic and phasic LC firing in an oddball detection task led to the hypothesis that tonic firing promoted distractibility or exploration-related behaviors, while phasic bursts promoted focused exploitation of learned behavioral strategies [5]. The exploration/exploitation hypothesis continues to shape interpretations of LC manipulations. During stress, for example, Valentino and van Bockstaele [6] propose that corticotrophin releasing factor (CRF) elicits heightened LC tonic firing and arousal, engaging scanning behavior, while opioid inputs promote phasic LC firing permitting termination of stress and maintenance of on-going behaviors. A contrasting theory emphasizes the role of neuromodulation in reconfiguring neural networks and elaborates a role for LC phasic activation in resetting networks to meet unexpected challenges [7].

The experimental challenge of recording from LC, located in the pons adjacent to the fluid-filled fourth ventricle, has meant that observations of LC firing patterns, and hence our understanding of their origins and significance, have been limited to a small number of neurons. However, recently, larger scale recordings are suggesting a more nuanced activity profile for LC neurons with high levels of variability. Synchronous LC activation, assumed to be the underpinning of phasic discharge, has been called into question as extensive recording sets have revealed that coincidental LC neuronal firing, either spontaneous or evoked, is rare [8]. The newest detailed investigations of LC neurons are consistent with a modular characterization of spatially distributed ensembles [9]. However, specifically, such LC neuronal subsets appear to activate transiently in varying groupings and to associate with multiple distinct cortical states beyond the original dichotomous aroused/non-aroused states [10].

The tools of optogenetics provide us with another way to interrogate the effects of LC temporal activation patterns on functional output. While, as we will see, evidence suggests it is unlikely that we can reliably drive LC neurons in vivo in a one-to-one ratio with light pulses, we can ask if different temporal patterns of optogenetic input activation at the same anatomical loci alter functional outcomes. Optogenetic inputs are considered phasic with durations ranging from a few hundred milliseconds to 10–20 s typically terminating with silence of longer duration. Transient activation on either the millisecond or seconds scale seems to lead to similar outcomes (but see (4) for an exception). Tonic optogenetic inputs, typically characterized, last over minutes.

The present perspective examines a set of pioneering LC optogenetic studies that have primarily used various tonic (sustained over minutes) and phasic (intentionally interrupted on a scale of seconds) temporal activation protocols to gain insights into the mediation of LC functions. We review these studies both with a view to the lessons learned from each and to the identification of important future directions. We are not reviewing studies using chemogenetic methods, despite their considerable interest, since there is no control of temporal firing patterns, at present, using these approaches.

(1) Phasic and tonic LC patterns both contribute to arousal in mice [11].

**Figure 1:**
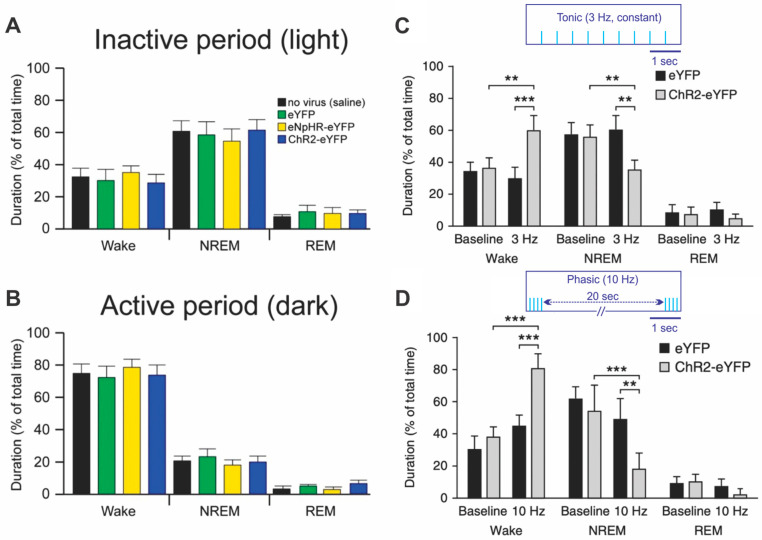
Effects of LC activation patterns on sleep-wake arousal. (**A**,**B**) reflect the normal percentages of awake, non-REM (non-rapid eye movement) sleep and REM (rapid eye movement) sleep over a one-hour sample in the inactive (**A**) or active (**B**) period of the light-dark cycle. The expression of light-sensitive inhibitory and excitatory channels did not alter the sleep-wake patterns relative to controls. (**C**,**D**) reflect the changes in duration of wake, non-REM and REM sleep when mice were given 1 h of bilateral tonic 3 Hz (**C**) or phasic 0.5 s/20 s activation (**D**) in the inactive period. ** *p* < 0.01 *** *p* < 0.001. Reprinted with permission from [11] 2010 Springer Nature BV.

The first study to explore the effects of phasic and tonic optogenetic LC activation was that of Carter et al. [11]. Their experiments characterized the natural durations of sleep and waking in mice in their inactive period and the role of LC in initiating transitions from sleep to waking. The inactive period was most sensitive to optogenetic LC activation. Transitions were defined based on electroencephalography (EEG) for non-rapid eye movement sleep (non-REM) and electromyography (EMG) for rapid eye movement sleep (REM). See Figure 1 for normal and LC-modulated arousal patterns averaged over a one-hour period. The expression of light-sensitive inhibitory and excitatory channels in LC neurons did not alter arousal patterns relative to no light controls. Tonic activation at 3 Hz increased waking duration and reduced non-REM duration. Phasic activation at 10 Hz for 0.5 s/20 s increased waking duration and non-REM duration to levels seen in the active period. The half second of 10 Hz every 20 s would have resulted in 150 LC action potentials in a 10 min period while the 3 Hz tonic pattern would have generated 1800 LC release events in the same period if optogenetic pulses faithfully drove LC neurons. (3) suggests faithful driving is unlikely at higher rates. On the other hand, older electrical LC experiments provide evidence for increases in noradrenaline (NA) release with phasic input when compared to tonic input, if both are matched for pulse numbers over time [12]. Unfortunately, we do not know the relationship between optogenetic LC activation patterns and NA levels in any target area. 

When given 5 h of continuous 3 Hz tonic or 10 Hz phasic activation in their inactive period, mice accommodated to 3 Hz tonic activation. Cumulative arousal patterns were unchanged from their normal inactive periods. Accommodation did not occur with phasic LC input over 5 h, consistent with stronger effects of the phasic pattern on arousal. 

Opposite effects on motor activity with phasic and tonic LC activation were seen during a 10 min wakefulness sample taken in the 1 h inactive period. Mice receiving 3 Hz tonic LC input were more active, while those receiving phasic input were less active, than controls. Neither group avoided the center of the testing cage. These activity differences may relate to greater rearing as reported with phasic patterns in rats [13], but rearing was not assessed here. Neither group displayed anxiogenic behavior consistent with the results of McCall et al. discussed in (3) when lower tonic rates and similar phasic rates of LC activation were assessed for aversive responses in mice [14]. Anxiogenic behavior seems especially sensitive to tonic optogenetic LC input frequency. The Carter et al. outcomes suggest tonic and phasic LC patterns can both support active period arousal with the phasic pattern here associated with more arousal than the 3 Hz tonic pattern.

Brief periods of photo-activation were tested for their ability to induce a sleep–wake transition (change from lower arousal to higher arousal) during non-REM and REM states in the inactive period. A frequency–duration trade-off was revealed: lower frequencies required longer duration activation periods to evoke a sleep–wake transition, e.g., 2 Hz for 10 s and 8 Hz for 2 s both provoked non-REM sleep/wake transitions, consistent with the hypothesis that higher frequencies are more arousing. REM sleep/wake transitions required longer LC activations but showed the same trade-off pattern with frequency. These data argue that reaching a particular cortical NA concentration within a short period initiates sleep–waking transitions [11].

Higher frequencies (5–20 Hz) and durations of stimulation, ~20 s could generate 15 s behavioral arrests. The probability of behavioral arrest increased from ~33% to 100% as frequency increased from 5 to 10 Hz. The latency to arrest decreased with higher frequencies but arrest duration was constant at ~15 s. Increasing NA pharmacologically ameliorated behavioral arrests suggesting they related to lower NA. 

Behavioral arrests were not seen in later studies [14] with similar frequencies but lower laser power (20 mW vs. 10 mW). If arrests are related to NA drops, the technologies to detect small variations in NA levels are now available [15]. Behavioral orienting, an interruption in on-going behavior tied to LC bursts, might be related to optogenetically-evoked behavioral arrests but it is not yet clear that arrests are physiological events.

While the arrest data suggest pauses in release might occur with brief high frequency bursts, direct microdialysis NA measurements in Carter et al., 2010 [11] revealed a fall in NA in the medial prefrontal cortex (mPFC) with 10 min of tonic 10 Hz LC activation. The NA decrease was similar to that seen with 10 min of optogenetic LC inhibition (in Carter et al., 2010 [11] supplementary figures). This suggests that sustained 10 Hz LC optogenetic activation leads either to NA exhaustion in terminal fields or possibly to LC silencing as reported later by Quinlan et al. in rats [16]. Thus, this initial study highlights the future value of recording LC activity during optogenetic activation and of assessing NA levels in target structures.

Although all behavioral experiments were carried out with bilateral LC optical fibers of ~200 microns, unilateral 3 Hz tonic LC activation for 1 h was employed to investigate the spatial extent of *c-fos* activation in LC, an index of cell firing. Consistent with the small size of mouse LC, they found *c-fos* activation throughout the ipsilateral LC’s anterior-posterior extent. Sixty-five percent of LC cells were reactive for *c-fos* on the ipsilateral side. On the contralateral side ~30% of LC units were *c-fos* reactive. This appears consistent with a report that ipsilateral electrical LC activation recruits contralateral LC firing [17]. However, a direct anatomical link between the two LCs is not known and 1 h of LC light activation would permit multiple indirect pathways to be recruited. What are the lines of communication? Control brain *c-fos* was 17% in both nuclei without LC light activation. It will be of interest to assess ipsilateral and contralateral *c-fos* patterns in other unilateral activation studies. LC neurons may have been active below the recruitment criteria for *c-fos* expression [18], but the data suggest tonic 3 Hz light pulses recruited two-thirds of ipsilateral LC neurons during the 1 h period.

Lesson learned: The Carter et al., 2010 experiments suggest optogenetic LC inputs have functional effects that depend on circadian rhythms and that exhibit frequency-duration trade-offs. Brief phasic activation with intentional pauses, has both common and distinct outcomes in comparison with tonic activation. Longer tonic optogenetic activation at higher frequencies can reduce LC NA output in target areas. Unilateral optical activation may recruit modest contralateral LC *c-fos* activation.

(2) Long-term behavioral plasticity occurs with tonic and phasic optogenetic activation paired with sensory stimuli in rat hindbrain and forebrain respectively [19,20].

Two studies were the first to report enduring changes in network behavior as a function of repeated optogenetic LC pairing with input. Hickey et al., 2014 [19] used a unique pattern of continuous optogenetic light over 60 s preceding thermal nociceptive input that peaked 5 s after light activation ended. They reported a long-term (hours long) suppression of thermal nociception with three repetitions at 8 min intervals. Their continuous light protocol of 60 s seems to most closely mimic tonic activation.

Martin et al., 2015 [20] found long-term (over days and weeks) effects of repeated phasic pairings of LC optogenetic activation (500 ms of optogenetic pulses at 20 Hz coincident with a 500 ms tone) both on frequency coding in auditory cortex and on long-term ability to detect the paired frequency. Martin et al. [20] identified a novel mechanism of LC support of the long-term encoding changes they observed. 

**Figure 2:**
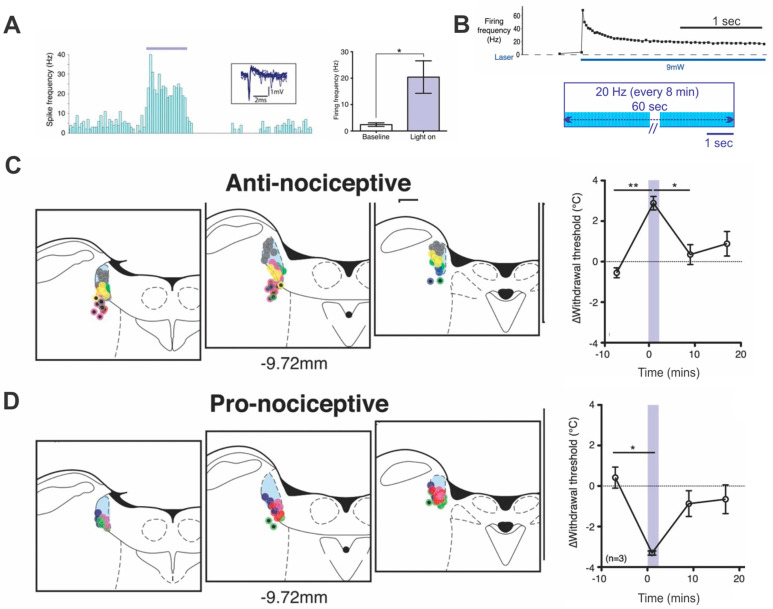
Regional LC optogenetic activations recruit sub-ensembles leading to differential behavior. (**A**) LC unit activity with a 20 s continuous 30 mW blue light. Note pause after activation. Mean firing rate of 20 Hz shown graphically for 5 rats. (**B**) Continuous blue light (9 mW) for 60 s was used in the nociceptive studies. Rapid adaption after initial peak resulted in maintained firing at ~20 Hz as shown in the cartoon. Long-term anti-nociception occurred with the LC light followed by a heat peak at 5 s and three repeated pairings each at an 8 min interval. (**C**) Anti-nociceptive locations identified in 7 rats with analgesic responses. Different rats are indicated by different color dots and dark core dots represent subcoerulear locations. Graph indicates increased threshold for heat pain-induced paw withdrawal at dorsal locations. (**D**) Pro-nociceptive locations in 4 rats showing increased pain sensitivity. Different rats are indicated by different color dots and dark core dots represent subcoerulear locations. Graph indicates decreased threshold for heat pain withdrawal at ventral LC sites. * *p* < 0.05; ** *p* < 0.01. Adapted with permission from [19] 2014 Creative Commons Attribution-Non-Commercial 4.0 International Public License (CC BY-NC 4.0).

The initial optogenetic investigations of LC modulation of nociception [19] highlighted a spatial selectivity of LC ensemble subsets vis-à-vis nociception that had not been previously appreciated. See Figure 2. Dorsal LC fiber optic placements produced pro-nociception while anti-nociception occurred at more ventral placements. The subsets overlap, however. These results are consistent with the newer understanding of LC as containing functionally distinct but intermingled LC ensembles innervating different targets [9,21,22]. This spatially more heterogenous LC organization indicates that in addition to any broadcast influences, LC ensembles can support targeted outcome mediation. See also, for example, the work of Usematsu et al. [23]. In the present Perspective we are suggesting that variation in temporal LC input patterns is a second source of target and functional heterogeneity. 

In the nociceptive experiments, sustained LC light on for 60 s, rather than pulses, was used for 2 reasons: (a) it produced higher firing rates with less laser power, rates were close to those nociceptive studies using LC electrical stimulation to modulate pain but were less toxic to LC neurons and (b) only about half of the in vitro LC neurons tested responded to 20 ms pulses at 20 mW, but all were recruited to fire by continual light at 9 mW. The continual light input produced a high initial peak rate followed rapidly by accommodation and a lower LC maintenance rate at ~20 Hz. Using this paradigm, Hickey et al. [19] reported that three repeated light-on activations at the same anti-nociceptive site could extend LC-induced thermal analgesia for several hours.

The ability of repeated pairings of sensory input with LC depolarization to result in enduring functional change was similarly reported with tone-LC pairings [1]. In these experiments both 20 Hz electrical LC activation and 20 Hz optogenetic LC activation were used. Both stimuli lasted 500 ms and were co-extensive with the 500 ms tone that was presented. Outcomes appeared similar. In an auditory cortex, there were two stages in the ‘re-tuning’ of auditory neurons with repeated tone-LC pairings. First, there was activation of an auditory neuron by a broader range of frequencies, followed by sharpened tuning to the LC-paired frequency. Cortical retuning depended on continued auditory cortex alpha-1 receptor activation suggesting that LC support of the altered tuning was occurring. Remarkably, after LC depolarization pairing with a tone, LC neurons now began to show EPSPs evoked by that tone and produced tone-evoked LC spikes, not previously seen. The control of LC firing by tone presentations and LC depolarization was prevented by an NMDA-receptor antagonist infused into LC during pairing. Thus, plastic changes in LC circuitry and in LC responses to sensory input acted to maintain the new sensory tuning observed in the auditory cortex. These changes were long-lasting, could influence behavior for weeks and required tone-evoked LC activation. In addition to expressing NMDA receptors, rodent LC has spines similar to those in cortical neurons [24,25]. The contribution of LC spines to NMDA-receptor mediated long-term LC plasticity remains to be investigated. The mechanism supporting enduring analgesic effects with repeated heat pain + LC pairings in Hickey et al. was not probed. While target structure changes are typically hypothesized to support LC-induced plasticity (e.g., [26]), the auditory tuning experiments suggest that changes in LC responses to paired input itself may contribute. 

Hickey et al. [19] confirmed the LC location of their optical fibers by measuring blood pressure. LC activation placement was identified by a significant lowering of arterial blood pressure at higher laser power (see Figure 2). This sympathoinhibitory effect of LC activation remains to be understood in more detail, although LC efferent connectivity is sufficiently complex to support such an outcome [27]. It is of interest that both 60 s and 500 ms of LC activation were effective in inducing enduring behavioral plasticity. This is consistent with other evidence that LC-NA outputs operate on multiple time scales ranging from milliseconds to hours [28]. 

Lessons learned: Optogenetic activation of LC can recruit different dominant neuronal subsets depending on fiber optic spatial location in rat LC. Repeated LC activation with sensory input can generate long-term functional change in both forebrain and hindbrain structures. Long-term changes induced by temporal pairing of LC and forebrain target structure activation may depend on bi-directional target-LC communication and be supported by NMDA-receptor induced plasticity in LC itself.

(3) Tonic activation patterns are selectively associated with anxiety and aversion in mice and optogenetic input at 5 Hz does not reliably predict LC firing in vivo [14].

**Figure 3:**
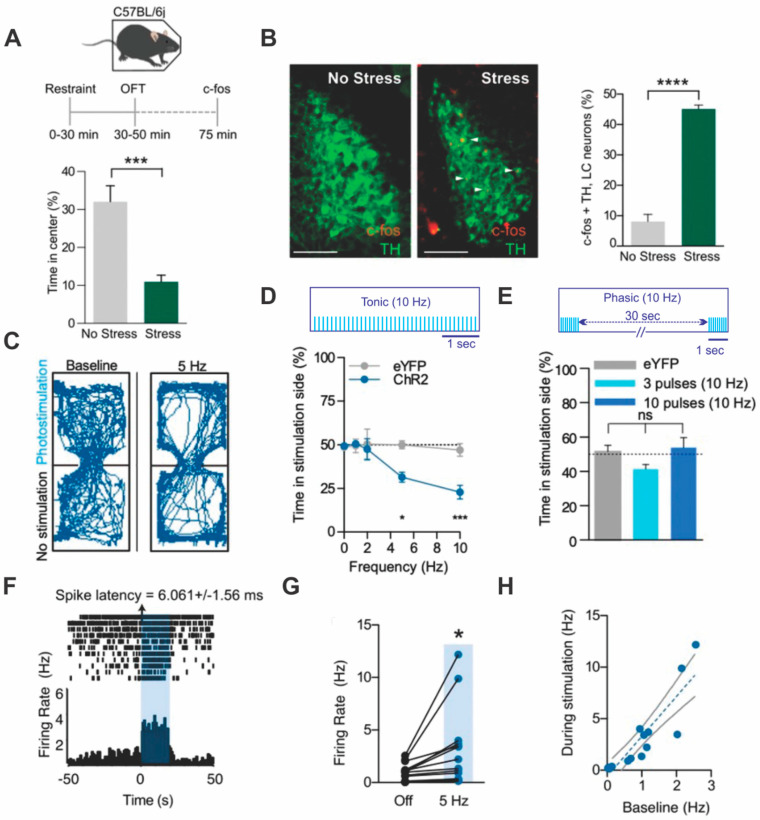
Tonic LC optogenetic stimulation induces anxiety and aversion despite heterogeneous LC cell firing. (**A**) Restraint stress was given to C57bl/j6 mice for 20 min before a 10 min open field test (OFT). Stressed mice spent less time in the center. (**B**) Locus coeruleus (LC) *c-fos* was examined in both no stress and stress groups. Red *c-fos* cells are among green tyrosine hydroxylase-reactive (TH) cells in a stressed mouse in the right image. Stress-induced LC *c-fos* activation of ~45% is quantified in the adjacent graph. (**C**) An illustration of place avoidance during a 20 min test with LC activation at 5 Hz when in the photo-stimulated chamber. Note center avoidance in both chambers. (**D**) The 10 Hz tonic pattern cartoon. Time in photo-stimulated side across tonic frequencies. Further, the 5–10 Hz tonic activations produce avoidance. (**E**) The 10 Hz 10 pulse phasic pattern cartoon. The 10 Hz phasic patterns do not produce avoidance in the same 20 min test. (**F**) An identified LC neuron histogram with repeated 20 s tonic 5 Hz light pulses indicated in blue. Repeated trials begin at the top of the raster display. Latency to first spike after light averages ~6 ms. Decreased baseline activity with repeated optogenetic activation trials is evident. (**G**) Firing rates at baseline and for light activation for 16 neurons using a 5 Hz 20 s train. Higher firing units were also recruited on repeated photo-stimulations. (**H**) The relation between baseline and light activated firing rates for the 5 Hz 20 s trains (r = +0.89). * *p* < 0.05; *** *p* < 0.01 **** *p* < 0.001. Adapted with permission from [14] Creative Commons Attribution-Non-Commercial 4.0 International Public License (CC BY-NC 4.0).

McCall et al. [14] used optogenetics to study the role of LC in stress-related behavioral change. They demonstrated that 20 min of restraint stress activates ~45% of LC neurons as indexed by *c-fos* (see Figure 3) and induces open field center avoidance. Tonic 5 Hz LC light activation for 20 min can replace restraint stress in leading to subsequent open field center avoidance. Anxiety defined as less time spent in the center of an open field was induced by 5 Hz tonic LC activation and prevented by prior administration of a β-adrenoreceptor antagonist.

Tonic 5 Hz light activation and higher frequencies also induced place avoidance for mice given 20 min to move spontaneously between a light activation and a no light activation chamber. Phasic LC light patterns resulted in the same amount of time in both chambers as was seen with controls (see Figure 3), unlike higher tonic patterns. This difference in real time compartment aversion argues for a unique role of higher tonic, rather than phasic, LC input activation in engaging stress-related behavior. It would be of interest to know the levels of NA associated with these patterns. 

The pharmacological experiments revealed that, unlike the anxiety effects seen in open field and elevated zero maze testing that were blocked by a beta-adrenoreceptor antagonist, real time place aversion was blocked by an alpha-1, but not by a beta-adrenoceptor, antagonist. Conditioned place aversion was also induced by pairing tonic 5 Hz light with a specific chamber for 30 min on 2 days. Chamber choice on the 3rd day revealed a conditioned place aversion.

To examine the responses of identified single LC neurons in vivo to 5 Hz light activation McCall et al. [14] measured responses to 5 Hz light pulses for multiple 20 s blocks under anesthesia. See Figure 3. None of the 16 neurons isolated fired at exactly 5 Hz over the 20 s period (averaging ~0–12 Hz as shown in Figure 3). The majority averaged under 5 Hz while several fired at frequencies from 10–20 Hz. For individual neurons with repeated activation, baseline firing decreased over time so that the light activation signal itself would have become more distinct (e.g., raster example in Figure 3). However, in addition, LC neurons not initially responsive to the LC light pulses were recruited to participate with repetition. The recruited neurons had higher spontaneous activity and higher firing rates during light activation. Across the original and expanded set of neurons (total *n* = 16), there was a +0.89 correlation between average baseline firing rate and light-activated firing rate. While more excitable neurons were recruited with repeated LC activation, it is not clear by what mechanism recruitment occurs. Excitatory post-synaptic potentials between LC neurons are not reported in vitro and electrotonic coupling is thought to be important primarily in developing LC. LC co-incident firing is rare in adult rodents in vivo [8]. The latency of the first action potential after a light pulse was <10 ms suggesting direct light depolarization of initial light-activated neurons.

In follow-up experiments, LC was excited by activating light channels in corticotrophin releasing hormone (CRH) neuron terminals projecting to pons from the central amygdala nucleus (CEA). The latency of an LC action potential after the light pulse for this input ranged from 200–600 ms suggesting indirect effects. While LC excitation via CRH would be expected to be slower due to G-protein receptor coupling, a recent tracing study of CEA inputs to LC argues that they contact parabrachial neurons rather than LC neurons directly [29]. This could account both for the long latencies and for the bidirectional effects on LC firing observed by McCall et al. As in the directly transduced LC neuron experiments, increased firing rates did not match the light input frequency (10 Hz). In the CRH experiments, a substantial LC neuronal subset decreased firing, rather than increasing firing with the excitatory light input. While decreases might be related to LC neurons generating lateral inhibition, they were not seen with direct LC light activation. Anxiety was generated by CRH optogenetic LC activation despite the mixed profile of LC responses.

Lessons learned: Tonic, but not phasic, optogenetic LC input induces anxiety and aversion. Optogenetic activation of LC in vivo does not produce one-to-one driving of LC neurons at higher frequencies, however repeated LC activation recruits additional neurons not initially light-activated.

(4) Optogenetic patterns designed to mimic LC novel open field firing replicate functional effects of exposure to a novel open field in mice. Repetitive phasic optogenetic LC activation paired with weak sensory input below the arousal threshold in anesthetized rats generates cognitively important cortical priming [30,31].

Takeuchi et al., 2016 [30] examined LC and ventral tegmental area (VTA) dopamine cell firing in awake mice during 5 min of novel environment exposure. This study has three features of interest. (1) It demonstrated a unique LC role in novel environment encoding. (2) LC firing associated with novel environment exposure was characterized. (3) An optogenetic mimic of novel environment LC firing produced the same functional result as novel environment exposure.

**Figure 4:**
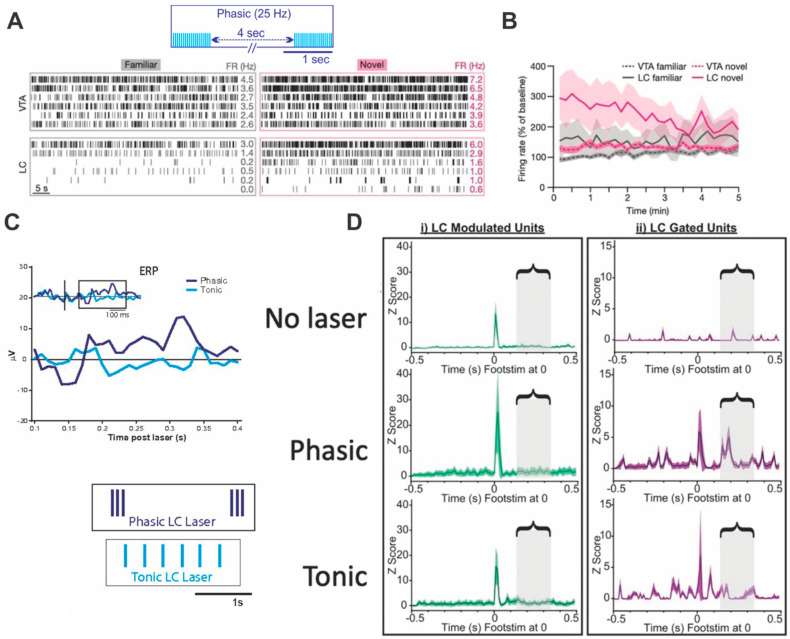
Phasic LC activation produces novelty-related memory extension and mimics sensory salience. (**A**) Ventral tegmental area (VTA) neurons and locus coeruleus (LC) neurons firing in a familiar versus novel environment are shown. Corresponding raster lines in the two settings are the same unit sampled for 1 min. The cartoon shows the phasic optogenetic pattern used to mimic novel environment firing of LC neurons. (**B**) The average of 15 neurons from 5 mice over the 5 min period in the two environments. LC units show habituation in firing rate. Reprinted from [30] 2016 Springer Nature BV. (**C**) Averaged event related potentials (ERP; *n* = 50 events) from the medial prefrontal cortex. Response times locked to phasic LC onset show N1- and P3-like components not seen with the tonic LC pattern. Cartoons show phasic and tonic patterns matched for number over 1 s. (**D**) Hindlimb evoked responses to a weak shock without LC photoactivation, with phasic LC photoactivation and with tonic LC photoactivation. Green lines represent early latency hindlimb responses that were always present to shock but were modulated by concurrent LC activation. Purple lines represent early and longer latency responses that were seen in nearby somatosensory neuron populations only when LC was concurrently activated (LC gated units). Adapted with permission from [31] 2018 PNAS.

In the novel environment, LC showed an initially higher activity with significant habituation. This was not true for VTA which had higher activity but no discernable habituation (see Figure 4). Post-encoding exposure to the novel environment extended spatial memory. To mimic the LC novelty firing pattern with optogenetic pulses, Takeuchi et al. [30] quantified novel environment firing patterns over 5 min for identified LC neurons. Bursts, defined as spikes 60 ms apart ending with a pause of 160 ms, made up ~25% of LC action potentials. The burst frequencies ranged from 15–28 Hz with a range of 0–80% of spikes in bursts for different neurons. The average firing rate over 5 min was ~2 Hz with a range 0.23–6 Hz. See Extended Data in the Takeuchi paper. Given the habituation pattern seen in Figure 4 LC firing in a novel environment is likely associated with bursts at early timepoints followed by slower irregular firing, although, as described, bursts were quite variable.

Using the LC unit data from freely behaving mice in the 5 min novel open field, the researchers attempted to mimic its effects with their optogenetic pattern. The optogenetic pattern did not have the habituating profile of LC natural firing. However, they recorded the unit activity produced by the optogenetic pattern under anesthesia. The experimenters had chosen a phasic optogenetic pattern of 20 pulses (18–19 mW) of 25 Hz/5 s (~1 s on/4 s off) for 5 min (see Figure 4). They observed burst firing rates in their light activated neurons ranging from 15–25 Hz similar to those in naturally recorded bursts. Light activated neurons were classified as those having spikes within 15 ms of a light pulse and on at least 1/3 light pulses. In their phasic activation optogenetic protocol, all spikes recorded under light anesthesia occurred in bursts. This differed from the natural pattern in which 25% of LC potentials occurred in bursts. Critically, however, the optogenetic burst pattern given post-encoding extended spatial memory as did the 5 min of novel open field exposure suggesting that even unphysiological input patterns may capture real parameters of LC function.

The Vazey et al., 2018 [31] study also has multiple features of interest. They made (1) direct comparisons of phasic and tonic LC activation effects on cortical indices of attention and salience and (2) they discovered that phasic activation reproduces two indices of network priming previously described in cognitive studies in humans and associated with the processing of novel and salient stimuli [31]. In Vazey et al. the phasic pattern was three pulses at 12 Hz/5 s, mimicking natural firing patterns. The tonic activation was at 3 Hz for ~1 s, also a natural tonic frequency. Although this is a short tonic duration relative to the other experiments discussed, the number of pulses/time was identical for tonic and phasic patterns, such that the burst feature of phasic activation is highlighted. Under their anesthetic conditions, arousal did not occur with either the tonic or phasic pattern. Thus, the modulation observed was unrelated to arousal *per se* and assumed to reflect selective attentional effects of LC activation.

The phasic pattern on its own triggered an evoked cortical event detected with 50 trials of averaging in mPFC (see Figure 4) and a similar local evoked field potential in primary somatosensory cortex (S1). These LC evoked events had an N1-like component at ~130 ms and a P3 component at ~330 ms. Thus, repeated phasic LC activation recapitulated orienting- and attention-associated cortical events described in the cognitive literature. The evoked signatures were not linked to neuronal firing in the cortical substrate. They likely prime neuronal substrates for generating salience activity in response to sensory input. Broadening of spatial and temporal cortical unit responses to hindlimb input are seen in their subsequent experiments.

When a weak shock to the hindlimb was followed by phasic or tonic LC activation, both LC patterns enhanced the short-latency foot shock evoked response, termed the LC-modulated response (see Figure 4) in hind limb neurons. However, late response components did not appear in these neurons.

However, with LC activations, a new response population appeared, termed the LC-gated responses. Now short-latency foot shock sensory responses occurred in somatosensory neurons that had not previously produced action potentials to the foot shock, reminiscent of auditory neurons that did not show frequency-tuned firing unless tones were paired with transient LC activation [20] as discussed in (2). The early component of these novel short latency responses was promoted equally by phasic and tonic LC activation. However, phasic activation also promoted long-latency responses in time windows corresponding to the times of the event related potentials previously associated with phasic LC input activation, particularly N1.

Thus, both phasic and tonic LC activation enhanced the strength of the original response and recruited a larger spatial representation of the foot shock event by bringing unresponsive somatosensory neurons to threshold. Additionally, phasic LC activation added a temporal salience signature by recruiting late responses in the new larger representation, see Figure 4.

Lessons learned: Phasic LC activation is associated with response to novelty. Optogenetic patterns mimicking LC firing in a novel environment produce the memory-extending effects of post-training novel environment exposure. Phasic LC activation timed to specific stimuli mimics sensory salience in experiments that dissociate attention from arousal. Repeated phasic LC activation not tied to stimuli supports the appearance of N1 and P3 activity. Tonic LC activation makes partial contributions to sensory salience but does not to contribute to these field potential signatures of attention.

(5) Differential effects of phasic and tonic activation patterns in rats can be understood in terms of differential recruitment of output microcircuits [13].

Working with rats and using bilateral activation of LC, Ghosh et al., 2021 [13] compared the effects of phasic and tonic LC activations from the same sites on spontaneous and learned behaviors. In the open field, phasic activation (10 Hz, 10 pulses/30 s) or tonic activation (10 Hz) increased rearing duration suggesting both inputs enhanced exploration, while 25 Hz tonic activation increased freezing suggesting stress, as seen earlier with lower frequencies in mice [14].

The differences in stress-related optogenetic frequencies with lower frequencies inducing stress in mice (McCall et al., 2015 [14]) and only higher frequencies being effective in rats (Ghosh et al., 2021 [13]) may reflect a species difference or may relate to the spatial extent of LC activation. In rats, Hirschberg et al., 2017 [32] used chemogenetics to activate LC and reported increased anxiety with LC firing ~15 Hz. In Ghosh et al. [14] LC firing at ~15 Hz was seen with 15–30 Hz optogenetic pulse activation in urethane-anesthetized rats. Thus, stress LC frequencies for rats may simply be higher than for mice. Alternatively, the ability of optical fibers in mice to reach all of LC, in contrast to the situation in rats, where fibers cover only a portion of LC may be important for frequency-related stress differences. The open field behavior testing in Ghosh et al. in rats was carried out over days in a within-subject design, so fiber optic placement was identical, only temporal patterns were altered.

**Figure 5:**
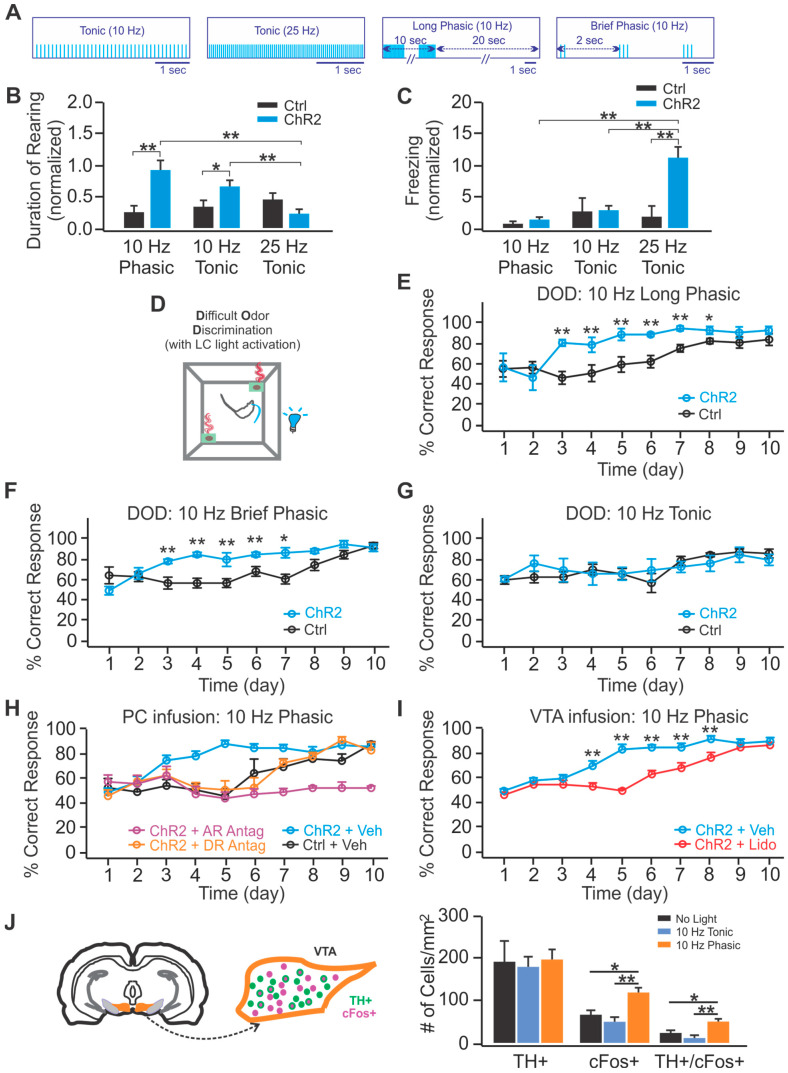
Phasic, but not tonic, LC activation improves odor discrimination learning via selective VTA activation. (**A**) The four blue light pulse patterns used to activate LC neurons. (**B**) Increases in rearing duration during a 10 min open field test with 10 Hz phasic or 10 Hz tonic LC activation relative to controls. (**C**) Increase in open field freezing behavior with 25 Hz tonic LC activation. (**D**) The odor discrimination task with blue light activation. The rat was discriminating two odors (octanol and heptanol in a 60:40 versus a 50:50 mixture; 0.001% concentration) for a cereal reward. (**E**) Ten pulses @ 10 Hz/30 s (long phasic) resulted in rats reaching criterion on Day 3 rather than Day 8. (**F**) Three pulses @ 10 Hz/2 s (brief phasic) also resulted in rats reaching criterion on Day 3. (**G**) Ten Hz tonic light activation did not enhance acquisition. (**H**) A dopamine-receptor antagonist in olfactory cortex (piriform cortex) prevents the phasic light-mediated learning acceleration but not the final discrimination. An adrenoceptor antagonist cocktail in the piriform cortex prevents acquisition of the difficult odor discrimination. (**I**) Lidocaine infusion in the ventral tegmental area (VTA) prevents the acceleration of odor discrimination acquisition but not discrimination itself. (**J**) *c-Fos* examination of VTA reveals similar numbers of VTA dopaminergic neurons (TH+) examined across groups. *c-Fos* activation by odor + LC light differs from no light controls with the 10 Hz phasic pattern, but not with the tonic 10 Hz pattern. Dopaminergic neurons of VTA are significantly more activated by odor + phasic LC activation. * *p* < 0.05; ** *p* < 0.01. Adapted with permission from [13] 2021 Creative Commons Attribution-Non-Commercial 4.0 International Public License (CC BY-NC 4.0).

Phasic activation at 10 Hz, 10 pulses/30 s, or at 10 Hz, 3 pulses/2 s were both effective in accelerating acquisition of a difficult odor discrimination, see Figure 5. Drug infusions in olfactory (piriform) cortex revealed, as previously known [33], that acquisition of the difficult odor discrimination was prevented by a mix of adrenoceptor antagonists. However, a dopamine receptor-antagonist infused in olfactory cortex prevented enhancement but not acquisition suggesting dopamine in olfactory cortex was responsible for learning acceleration in this paradigm. VTA lidocaine infusion also prevented the LC phasic activation-induced enhancement of learning rate. Consistent with these observations, *c-fos* activation of VTA neurons containing TH was enhanced by phasic, but not by tonic, 10 Hz light LC activation providing anatomical evidence that phasic and tonic 10 Hz light LC inputs differentially recruit dopaminergic VTA neurons, see Figure 5.

Pairing phasic activation with odor produced a conditioned odor preference and increased time spent with a light-associated odor, referred to as real time odor preference. The conditioning of an odor preference supports older hypotheses that LC activation can induce a positive valence [34,35].

Conversely, conditioning of an odor aversion was seen when 25 Hz tonic light activation was paired with odor. The conditioned odor aversion replicates place aversions seen earlier with tonic LC activations in mice [14]. For valence, phasic and tonic temporal optogenetic inputs generate dramatically different outcomes. Conditioned preferences and conditioned aversions could be blocked by infusing an adrenoceptor antagonist mixture in basolateral amygdala. This was not true of the real time odor preferences that likely rely on different circuitry.

Intersectional studies revealed that the positive valence-associated phasic light activated BLA neurons were marked by c-fos activation as projecting predominantly to nucleus accumbens rather than CEA. The same phasic pattern of LC activation also enhanced *c-fos* in VTA neurons projecting to nucleus accumbens, suggesting activation of parallel rewarding circuitries with phasic LC inputs.

**Figure 6:**
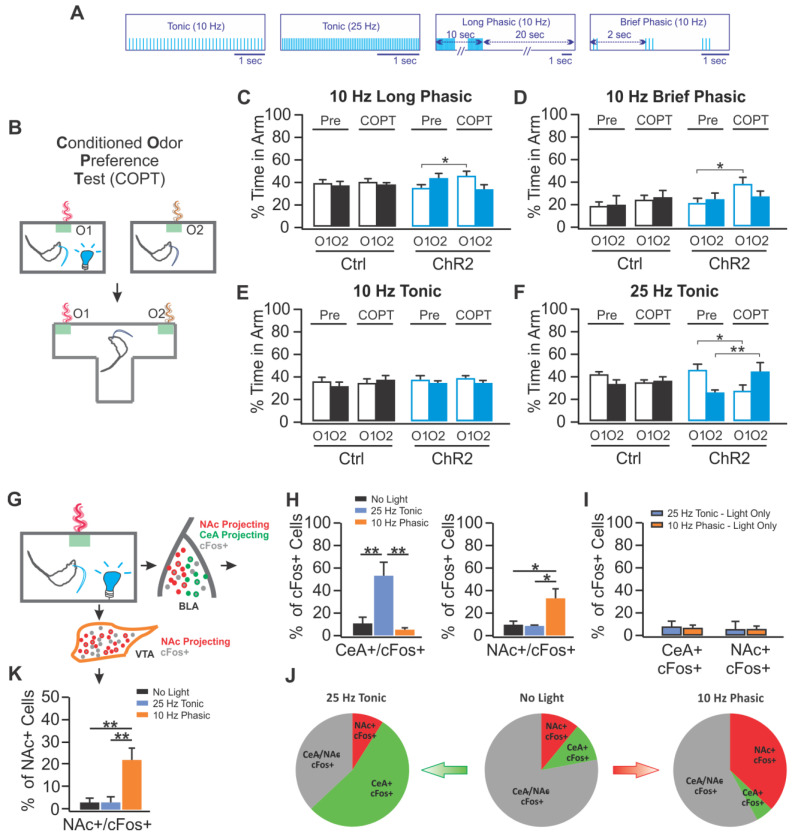
Phasic and tonic LC activations lead to differential valence odor learning. (**A**) The four blue light pulse patterns used to activate LC neurons. (**B**) Schematic for conditioned odor preference protocol. (**C**) A conditioned odor preference following odor pairing with long phasic LC (10 Hz for 10 s/30 s) (**D**) A conditioned odor preference following odor pairing with brief phasic LC (10 Hz for 300 ms/2 s). (**E**) No preference effect when pairing odor with 10 Hz tonic LC activation. (**F**) A conditioned odor aversion following odor pairing with 25 Hz tonic LC activation. (**G**). The protocol used for c-Fos studies. (**H**) The central amygdala (CeA) projecting neurons in the basolateral nucleus of the amygdala (BLA) were significantly activated by 25 Hz tonic LC activation paired with odor relative to no light and brief phasic LC activation controls. BLA neurons projecting to the nucleus accumbens (NAc) showed significant c-Fos activation relative to no light and 25 Hz tonic controls when odor was paired with phasic LC activation by 3 pulses of 10 Hz light/2 s for 10 min. (**I**) If only the phasic or tonic LC activation was given for the same period without a novel odor present, there was no difference in c-Fos neurons activated by the two patterns and relatively few c-Fos neurons were observed. (**J**) Pie charts showing the proportion of CEA (green) and NAc (red) projecting neurons as a fraction of the total set of c-Fos neurons (grey + green + red) observed in BLA with 25 Hz LC tonic + odor, no light + odor, and brief 10 Hz phasic LC + odor. (**K**) NAc-projecting neurons in VTA showed increased c-Fos with brief 10 Hz phasic LC + odor relative to no light + odor and 25 Hz LC tonic + odor groups. * *p* < 0.05; ** *p* < 0.01. Adapted with permission from [13] 2021 Creative Commons Attribution-Non-Commercial 4.0 International Public License (CC BY-NC 4.0).

The conditioned aversion produced by 25 Hz tonic light pairing was associated with more *c-fos* activation of BLA neurons projecting to CEA rather than nucleus accumbens, see Figure 6. Thus, the blockade of conditioned preferences and aversions seen with BLA infusion of an adrenoceptor antagonist mixture occurs because of a mixture of appetitive and aversive microcircuitry in BLA recruited differentially by different patterns of LC light activation. It should be emphasized that phasic and tonic LC activation alone do not recruit BLA microcircuitry differentially unless they are paired with odor. This underscores LC’s functional role as a neuromodulator for exogenous events and further suggests the affective component of that neuromodulation is related to its temporal properties.

Lessons Learned: Phasic LC optogenetic activation in rats engages enhanced exploration, enhanced acquisition of a difficult odor discrimination and confers a positive valence when paired with odor. Tonic activation also enhances exploration but does not alter acquisition of a difficult odor discrimination or alter valence when set to the same frequency used for phasic activation. If frequency is sufficiently high, tonic activation has aversive properties, inducing freezing and conferring a negative valence when paired with odor. LC activation recruits the ventral tegmental area (VTA) to support enhanced odor acquisition. LC phasic and tonic activation recruit separate microcircuits in the basolateral nucleus of amygdala (BLA) to support positive and negative valence. These outcomes strongly suggest temporal patterns of optogenetic input at the same LC site engage differing output structures.

## 2. Future Directions

LC optogenetic input patterns result in different functional outcomes, in part at least, by recruiting different target structure microcircuits. This is clearly reflected in *c-fos* intersectional studies [13,36]. Differences in response to target receptor antagonists for stress effects produced with the same LC input frequency, as seen with place-associated anxiety and place-associated avoidance [14], also provide evidence for different target effects with the same LC placements. Previously it had been hypothesized that receptor recruitment differences, as a function of local NA concentration differences, would explain differences in functional outcomes in target structures. This idea is captured in the Glutamate Amplifies Noradrenergic Effects or GANE framework [26], which hypothesizes that glutamate activity in target structures amplifies local NA release to alter local concentrations. Local concentration differences differentially promote plasticity mechanisms that enhance salient information and suppress less salient events. However, in the case of anxiety, mediated by low affinity beta 1 receptors, and real place avoidance, mediated by higher affinity alpha 1 receptors, it is hard to understand how concentration differences would explain the pharmacological effects since they are produced by the same LC activation. It may be that different target structures are involved in anxiety and aversion or, if GANE is correct, that glutamatergic activity in the same target structure(s) differs for the two kinds of behavioral testing (anxiety and avoidance) such that local NA levels are different. Alternatively, anxiety and aversion may be influenced by differential release of NA and neuropeptides. Twenty-four h anxiogenic effects of 5 Hz optogenetic activation in mice have been related to galanin release with only acute effects associated with NA [37], although that does not explain the differential NA antagonist effects. Direct measurements of NA and neuropeptide output in target structures are required to evaluate these mechanisms.

The input pattern differences between phasic and tonic optogenetic activation, and the apparently wide tolerance for different phasic patterns as seen, for example, in Ghosh et al. [13] suggests the intermittent pauses in LC firing are significant for the functional outcome differences in plasticity, with, for example, bursts at 10 Hz recruiting VTA neurons and tonic activation at 10 Hz not recruiting VTA neurons. Pauses also appear critical for eliminating place-related stress in McCall et al. [14]. The differential effects of pauses may relate to a requirement for altering NA levels in order to re-engage Gαs (a G protein-coupled receptor that increases cAMP) or to other downstream effects of chronically sustained G protein-coupled receptor activation [38].

The optogenetic studies suggest a plethora of phasic and tonic activation input differences that argue against a black and white dichotomy between exploitation and exploration as the linchpin of phasic/tonic functional roles. While phasic input does promote learning about and responding appropriately to sensory stimuli such a functional role is necessary in both exploration and exploitation. Tonic activation too has a broad involvement in arousal and attention, while at higher frequencies it is a component of stress states, mediating some of their behavioral consequences and constraining exploration. Optogenetic LC phasic input repeatedly paired with specific sensory inputs accelerates long-term behavioral change, but stress-related tonic activity can also induce long-term behavioral change.

The new data suggest we might usefully revisit the issues of valence encoding. Other behavioral paradigms focused on decision making and behavioral effort, now highlighted as LC roles in primate studies, for example, should be developed to assess the role of causal optogenetic manipulations—see for example, Bari et al. [39].

Ideally, in future optogenetic input studies, we will concomitantly interrogate LC firing output in behaving rats. At present we are only inferring LC outputs. LC firing in vivo to optogenetic inputs appears to be highly variable except at low tonic frequencies and possibly with some phasic patterns. Measures of *c-fos* activation even with stress suggest only some LC neurons are strongly activated. Why is that? Bilateral activation seems preferable given normal LC firing patterns, but it is not clear from optogenetic studies when it is important. In our pharmacological activation studies, spatial maps in hippocampal CA1 and CA3 are only modified by bilateral, not unilateral, LC activation [40], although hippocampal NA-driven synaptic plasticity can be reliably seen unilaterally in dentate gyrus even with optogenetic activation [16].

Chemogenetic activation suggests LC neurons have self-regulating properties. Do they also shape responses to optogenetic input? Do afferents have a critical role in LC cell excitability as hypothesized by Li et al. (2016) [41]? Do excited and inhibited LC ensembles co-exist and add to the heterogeneity of LC functional output? What are the mechanisms by which LC neurons become recruited with repeated optogenetic activation? Does activation repetition enhance the gain of LC output as it might? What is the role of repeated pairings or repeated LC activation in long-term behavioral change? Bi-directional interactions between LC and its targets require more attention. The observation of reciprocal excitatory exchanges between LC and mPFC is an important example of possible top-down regulation of LC [42]. Besides the long-term alterations of auditory tuning [20], are there other instances in which bi-directional LC-target interactions are modified long-term via LC NMDA-receptor plasticity?

What features of LC optogenetic activation result in a failure of NA release? Ten Hz tonic photoactivation sustained over 10 min appears sufficient to reduce NA release and may induce LC silence over time [11,16]. The LC correlates of brief behavioral arrests seen with short high frequency photoactivation patterns are still not understood [11]. The differing role of LC in supporting inactive period and active period arousal states is also highlighted by optogenetic LC investigations [11] and requires more exploration. Would behavioral assessments in active and inactive periods make a difference to LC functional outcomes and why?

Particularly fascinating is the apparent recruitment of N1 and P3 potentials in rodents by phasic activation unrelated to specific sensory input [31]. What is the mechanism of these spatially and temporally broad priming effects of pulsatile NA release? Priming events widely dispersed across cortical regions may account for the increased BOLD signaling supported specifically by LC activation [43].

New findings in a rat model of human pretangle tau reveal that LC neuron health is significantly and differentially affected by multiple exposures to phasic and tonic LC patterns [44]. Multisession exposure in adulthood to the brief phasic pattern seen in Figure 5 results in improved spatial and olfactory cognition and in the maintenance of the LC axonal arbors normally lost over time in the pretangle Alzheimer’s Disease model [44]. Multisession exposure to the tonic stress-related pattern of Figure 5 does not help cognitive loss but results in enduring anxious and depressive behaviors and compromised LC neuronal health indexed by higher levels of an apoptotic marker.

## 3. Summary

Pioneering optogenetic LC activation experiments have introduced new paradigms. Temporal patterns of LC activation alter LC output in ways that have distinct functional consequences via recruitment, or not, of other cell groups such as VTA, and engagement, or not, of specific behavioral circuitry, as in the basolateral amygdala. The distinct effects of temporal patterns span the gamut from promoting positive to negative valence and promoting engagement or disengagement with the environment. Ideally, we will, in future, be able to relate these effects to concomitant largescale recording of LC neuronal activity and to sensitive neurochemical measures of temporally fluctuating LC NA, dopamine, neuropeptide and glutamate output. Consistent with a proposed role for LC in synaptic plasticity, the optogenetic experiments provide evidence that phasic activation of LC accelerates acquisition of difficult discriminations and increases memory duration for events encoded prior to LC activation. Top-down and bidirectional effects on LC activity have been highlighted in these experiments. Importantly, phasic LC activity alone may prime the brain for dealing with unexpected events while LC activation more broadly contributes to the increased salience and selection, or suppression, of exogenous input.

With the availability of improved recording and neurochemical technologies we will begin to understand the sources of these heterogenous temporal patterning effects. The promise that characteristic patterns of LC activity over time have causal roles in LC health and, hence, in brain health, reinforces the importance of clarifying the real-world regulation of LC temporal patterns and their functions.

## Data Availability

All data obtained from published sources.
